# The Interplay of Chromatin Landscape and DNA-Binding Context Suggests Distinct Modes of EIN3 Regulation in *Arabidopsis thaliana*

**DOI:** 10.3389/fpls.2016.02044

**Published:** 2017-01-09

**Authors:** Elena V. Zemlyanskaya, Victor G. Levitsky, Dmitry Y. Oshchepkov, Ivo Grosse, Victoria V. Mironova

**Affiliations:** ^1^Institute of Cytology and Genetics, Siberian Branch of the Russian Academy of Sciences (SB RAS), NovosibirskRussia; ^2^Department of Natural Sciences, Novosibirsk State UniversityNovosibirsk, Russia; ^3^Institute of Computer Science, Martin Luther University Halle-WittenbergHalle(Saale), Germany; ^4^German Centre for Integrative Biodiversity Research (iDiv) Halle-Jena-LeipzigLeipzig, Germany

**Keywords:** bioinformatics, transcriptional regulation, TEIL, ETHYLENE-INSENSITIVE3, ChIP-Seq, EIN3 binding site (EBS), position weight matrix, Gene Ontology

## Abstract

The plant hormone ethylene regulates numerous developmental processes and stress responses. Ethylene signaling proceeds via a linear pathway, which activates transcription factor (TF) EIN3, a primary transcriptional regulator of ethylene response. EIN3 influences gene expression upon binding to a specific sequence in gene promoters. This interaction, however, might be considerably affected by additional co-factors. In this work, we perform whole genome bioinformatics study to identify the impact of epigenetic factors in EIN3 functioning. The analysis of publicly available ChIP-Seq data on EIN3 binding in *Arabidopsis thaliana* showed bimodality of distribution of EIN3 binding regions (EBRs) in gene promoters. Besides a sharp peak in close proximity to transcription start site, which is a common binding region for a wide variety of TFs, we found an additional extended peak in the distal promoter region. We characterized all EBRs with respect to the epigenetic status appealing to previously published genome-wide map of nine chromatin states in *A. thaliana*. We found that the implicit distal peak was associated with a specific chromatin state (referred to as chromatin state 4 in the primary source), which was just poorly represented in the pronounced proximal peak. Intriguingly, EBRs corresponding to this chromatin state 4 were significantly associated with ethylene response, unlike the others representing the overwhelming majority of EBRs related to the explicit proximal peak. Moreover, we found that specific EIN3 binding sequences predicted with previously described model were enriched in the EBRs mapped to the chromatin state 4, but not to the rest ones. These results allow us to conclude that the interplay of genetic and epigenetic factors might cause the distinct modes of EIN3 regulation.

## Introduction

The gaseous plant hormone ethylene regulates numerous plant developmental processes and stress responses, including germination, seedling growth, sex determination, fruit ripening, senescence, abscission, plant–microbe interactions and abiotic stress responses (McManus, 2012). Such a diversity is due to the fine regulation of ethylene signaling, which is under control of complex interactions with ethylene unrelated signals. Unraveling this complexity with respect to both genetic and epigenetic components is one of the major objectives in ethylene biology.

Cellular response to ethylene starts with ER-localized ethylene receptors, which transmit the signal via a linear pathway to the TFs of EIL family – the primary transcriptional regulators of ethylene response (reviewed in Merchante et al., 2013; Cho and Yoo, 2015). EILs activate transcriptional cascades, including secondary ethylene response mediated by ETHYLENE RESPONSIVE FACTOR1 (ERF1) (Solano et al., 1998). In the *Arabidopsis thaliana* genome there are six *EIL* genes (*EIN3, EIL1-5*) (Guo and Ecker, 2004), but only EIN3 and EIL1 proteins function as primary transcriptional regulators in ethylene signaling and mediate most, if not all, plant responses to ethylene (Chao et al., 1997; Solano et al., 1998; Alonso et al., 2003; An et al., 2010). EIN3 and EIL1 influence gene expression upon binding to a specific nucleotide sequence in gene promoters. The consensus binding site was described for tobacco EIN3 homolog (TEIL) as AYGWAYCT motif, where Y and W represent C/T and A/T, respectively (Kosugi and Ohashi, 2000). EBSs with a certain similarity to TEIL motif have been proven *in vivo* and *in vitro* in the upstream regions of a number of *A. thaliana* genes, e.g., *ERF1* (Solano et al., 1998), *HLS1* (An et al., 2012), *PIF3* (Zhong et al., 2012), etc. The TEIL motif was also found significantly enriched in EIN3 binding regions revealed by ChIP-Seq in *A. thaliana* (Chang et al., 2013). The majority of ChIP-Seq derived EIN3 target genes, however, did not respond to ethylene treatment, which implies the existence of more complex regulation of EIL-mediated gene expression.

In plants EIN3/EIL1 activity and DNA binding capacity are modulated by a variety of ethylene unrelated co-factors (Zhu et al., 2011; An et al., 2012; Song et al., 2014). In general, epigenetic modifications are known to play essential role in tuning activity of different TFs (Filion et al., 2010). Accordingly, epigenetic regulation was reported for EIN3/EIL1-mediated gene expression. JAZ protein, a transcriptional repressor, which participates in jasmonic acid signaling, is capable of interacting with EIN3/EIL1, recruiting an RPD3-type histone deacetylase HDA6 to the complex (Zhu et al., 2011). HDA6 introduces epigenetic chromatin modifications, thereby inactivating EIN3. However, the role of epigenetics in primary ethylene response has not been explicitly explored.

Here, we explore this possibility and study if there are associations of the occurrence of EIN3/EIL1 binding sites and different chromatin states in *A. thaliana* as published by Sequeira-Mendes et al. (2014). Based on the combinatorial co-occurrence of 16 chromatin features and the GC content Sequeira-Mendes et al. (2014) distinguish nine chromatin states. States 1, 2, and 3 contain a high amount of active chromatin marks (e.g., H3K4me3, H3K4me2, or H3K36me3), and are typically localized around the TSS, in proximal promoter regions and in genes, correspondingly. The other six chromatin states contain a low amount of active chromatin marks. Specifically, chromatin states 8 and 9 are enriched in heterochromatin marks such as H3K27me1, H3K9me2, GC methylation, and H3.1, while chromatin states 4 and 5 are enriched in the repressive chromatin mark H3K27me3. States 4 and 5 are predominantly intergenic, states 6 and 7 are intragenic.

We processed publicly available raw ChIP-Seq data on EIN3 binding (Chang et al., 2013) and investigated the whole genome distribution of obtained EIN3 binding regions (EBRs). Next, we used the genome-wide map of chromatin states in *A. thaliana* (Sequeira-Mendes et al., 2014) to characterize all EBRs with respect to the epigenetic status. Subsequently, we implied GO enrichment analysis to identify functional peculiarities of genes harboring the EBRs related to certain chromatin states in their 5′ regulatory regions. Finally, we performed motif enrichment analysis to specify interrelations between genetic and epigenetic components. Based on the results, we assumed that there is the interplay of genetic and epigenetic factors, which might cause distinct modes of EIN3 regulation.

## Materials and Methods

### ChIP-Seq Data Analysis

Two publicly available raw ChIP-Seq datasets on EIN3 binding in 3-day-old *A. thaliana* etiolated seedlings (Chang et al., 2013) were taken from NCBI Sequence Read Archive (SRA)^1^. The datasets SRX216234 and SRX215430 represented data on 4-h ethylene treated plants at ethylene gas concentration of 10 μl/l and control plants with no ethylene treatment, correspondingly. Individual ChIP-Seq runs were pooled for each dataset.

The genome sequence of *A. thaliana* was retrieved from TAIR 10^2^. The ChIP-Seq reads were mapped to the reference *A. thaliana* genome with Bowtie v. 1.1.1 (Langmead et al., 2009). The Bowtie options were set to report only unique alignments with no mismatches (-n 0 -m 1 --best). Peak calling was performed with MACS v. 1.4.2. (Zhang et al., 2008). The parameters were set by default. Peak calling was performed for 4-h ethylene treated plants (dataset SRX216234) using ethylene untreated EIN3 ChIP sample (dataset SRX215430) as a control according to the procedure in the primary source (Chang et al., 2013). 2577 ChIP-Seq peaks of height at least seven were considered EBRs.

Since the standard procedure for ChIP-Seq data analysis according to the ENCODE3 standards^3^ requires the presence of an input DNA control sample, we applied an alternative pipeline to confirm the robustness of the results (Supplementary Data 1).

### Analysis of the Distribution of EBRs

Genome annotation data for *A. thaliana* was retrieved from TAIR 10^4^. The GFF table was used to retrieve the chromosomal positions of transcription starts/ends and intergenic spacers.

For the analysis of EBRs distribution relative to the gene structure, we kept 35176 transcripts of protein-coding genes. The EBRs positions were classified as in (Boldyreva et al., 2016) with the following modifications: “TSS” (overlapping with at least one of the gene TSS); “GENE” (all other regions overlapping with the gene bodies); “INTERGENIC” (the regions outside of the genes). To obtain an estimate of non-randomness of EBRs distribution relative to the gene structure, Monte-Carlo permutation test was applied as described previously (Boldyreva et al., 2016). As a result, Monte-Carlo test provided a set of three *p*-values, which reflected non-randomness (enrichment or depletion) of the number of EBRs mapped to each of three location classes.

To characterize the distribution of EBRs relative to TSS we used a subset of 19434 genes with annotated 5′ untranslated region (5′UTR). The frequency of EBRs occurrence at a certain position in [-1500; +100] upstream gene region was estimated as the fraction of genes hitting EBRs.

### Analysis of Chromatin States

To characterize EBRs with respect to genome functional topography we used the whole genome map of nine chromatin states in *A. thaliana* (Sequeira-Mendes et al., 2014).

To statistically evaluate the representation of EBRs in the domains of chromatin states 1/2/4 along the upstream gene regions and perform chromatin-specific motif search (see below), we intersected annotations of 2577 EBRs with those of the chromatin states and compiled three datasets of 418/734/760 continuous EBR fragments, which were strictly mapped to the corresponding chromatin domains.

### Monte-Carlo Permutation Test for Genome Tracks

To estimate non-randomness of the overlap between various tracks of genome regions – EBRs, the domains of the chromatin states, EBR fragments (see above), the tracks of upstream gene regions (see below) – we used Monte-Carlo permutation test as described in (Khoroshko et al., 2016). We used the ratio of the total overlap length to the total length of the permutated track as a measure of overlap between two tracks. This ratio was referred to as the *fraction of overlap*.

The tracks of upstream gene regions representing seven 500 bp long intervals of [-3500; +1] region and entire 5′UTRs were created as follows. For the set of 31614 transcripts with distinct TSS we compiled annotations for eight regions: [3500; -3000], [-3000; -2500], etc. up to [500; +1] and [+1; AUG] relative to TSS. These datasets were referred to as the 1st, 2nd, etc. up to the 8th. We removed from the first seven datasets all sequences that had any overlap with annotation of any transcripts. We filtered out from 1st, 2nd, etc. up to the 6th dataset all sequences that have any overlap with annotation of upstream regions in the ranges [-3000; +1], [2500; +1], … etc. up to [-500; +1], respectively. We removed from the 8th dataset (1) all fragments of genes from the starts to the end of translation and (2) any sequences overlapping [500; +1] region. In total, the 1st, 2nd, etc. up to the 8th dataset consisted of 3430, 4152, 5179, 6620, 8856, 12645, 18768, 15238 sequences, correspondingly.

### Gene Ontology (GO) Enrichment Analysis

Functional annotation of genes was performed using 6.8 version of DAVID tool (Huang et al., 2009)^5^ separately for two sets of genes, which were created as follows. For each EBR we defined a set of genes harboring the EBR in their [3500; +1] upstream regions and indicated if EBR overlapped with annotations of active chromatin states 1, 2, and 4. Next, we compiled two list of genes: (1) the genes with EBRs mapped to the domains of chromatin states 1 or 2, but not 4; and (2) the genes with EBRs mapped to the domains of chromatin state 4, but not 1 or 2. Finally, we removed duplications in each list and all the genes occurring simultaneously in both lists. The resulting lists of genes were referred to as ‘states 1 or 2’ and ‘state 4.’ The default whole genome background dataset of DAVID tool was used for GO enrichment analysis.

We used default annotation categories GO_TERM_ BP_DIRECT, GO_TERM_MF_DIRECT, and GO_TERM_MF_DIRECT to deduce the lists of GO terms for vocabularies of *biological processes (BPs), molecular functions (MFs)*, and *cellular components (CCs).* The significance of enrichment of GO terms was estimated by the EASE Score, a modified Fisher’s exact *p*-value (a built-in function of DAVID tool). The following thresholds were set to distinguish the robust GO terms for each list: (1) the fraction of genes belonging to GO term > 3% and (2) false discovery rate (FDR) < 0.05 (Benjamini-Hochberg correction, a built-in function of DAVID tool). Accordingly, we compiled a set of GO terms, which were robust for either ‘states 1 or 2’ or ‘state 4’ lists.

To compare fractions of involved genes for each GO term from the compiled set between two lists we applied the exact Fisher’s test of a 2 × 2 contingency table (**Table 1**), where ‘Criterion 1’ stands for ‘The list name,’ X1 and Y1 denote ‘states 1 or 2’ and ‘state 4,’ correspondingly; ‘Criterion 2’ stands for ‘GO term,’ X2 and Y2 – the numbers of genes belonging to the GO term or not, correspondingly. We applied Benjamini-Hochberg correction for multiple testing (Benjamini and Hochberg, 1995) to define robust GO terms enriched in one list in comparison with another. The false discovery rate threshold was set as FDR < 0.05.

**Table 1:** The conventional 2 × 2 contingency table used in analysis.

Criterion 1	Criterion 2		Totals
	X2	Y2	
X1	*A*	*B*	*A*+*B*
Y1	*C*	*D*	*C*+*D*
Totals	*A*+*C*	*B*+*D*	*A*+*B*+*C*+*D*

### Prediction of Putative EIN3 Binding Sites

To identify potential EBS we used the Position Weight Matrix (PWM) deduced by NLG approach of weight calculation (Levitsky et al., 2007) from the position frequency matrix for TEIL motif described in (Kosugi and Ohashi, 2000). The threshold of 0.91 for this PWM was chosen since it respected to enrichment of the potential EBS density in the control dataset in comparison with the whole genome (Supplementary Figure S1A), but still the False Positive rate was left on a permissive level (Supplementary Figure S1B). The control dataset was defined as upstream regions in the range from -1500 relative to TSS to the translation starts for 375 genes, which were earlier reported as ethylene regulated EIN3 candidate targets (list EIN3-R from Supplementary Data 1, Chang et al., 2013).

We applied the TEIL model to 16 sets of EBR fragments generated by mapping of 1152/760 EBR fragments related to chromatin states (1 or 2)/4 (see Analysis of Chromatin States) to previously described eight datasets of upstream gene regions (see Monte-Carlo Permutation Test for Genome Tracks).

To compare the occurrence of potential EBS in chromatin state 4 to the one in chromatin states 1 or 2 for eight pairs of upstream regions we applied Fisher’s exact test of a 2 × 2 contingency table (**Table 1**). In this table ‘Criterion 1’ stands for ‘Chromatin state,’ X1 and Y1 – for ‘states 1 or 2’ and ‘state 4,’ correspondingly; ‘Criterion 2’ stands for ‘Prediction of EBS,’ X2 and Y2 denote the total counts of TEIL matrix scores above and below the threshold, correspondingly. Since the computations were performed for eight datasets simultaneously, we applied Bonferroni correction for the significance level threshold of *p*-value < 0.05/8.

## Results and Discussion

### ChIP-Seq Derived EIN3 Binding Regions (EBRs)

To get a genome-wide view of EBRs we performed an analysis of publicly available ChIP-Seq data on EIN3 binding in 3-days-old *A. thaliana* etiolated seedlings (Chang et al., 2013). Namely, we used the data for *A. thaliana* Col-0 ecotype (1) treated by ethylene gas at 10 μl/l for 4 h and (2) without ethylene treatment. The latter dataset was used as a control. As only raw data were available, we performed mapping of raw reads and subsequent peak calling. To perform fine analysis of ChIP-Seq data, Bowtie alignment contained only uniquely mapped reads with no mismatches. Finally, we had 5285145 reads for the control dataset and 4118771 reads for the ethylene treated one. The mapping quality statistics is depicted in Supplementary Figure S2.

After peak calling with MACS (genome profile in WIG format in Supplementary Data 2) the maximal peak height value was limited by seven. As a result, 2577 peaks were mapped in chromosomes 1–5, which were considered EBRs. The majority of peaks (>95%) had the length below 500 nucleotides (Supplementary Figure S3).

### Distribution of EBRs

**FIGURE 1:**
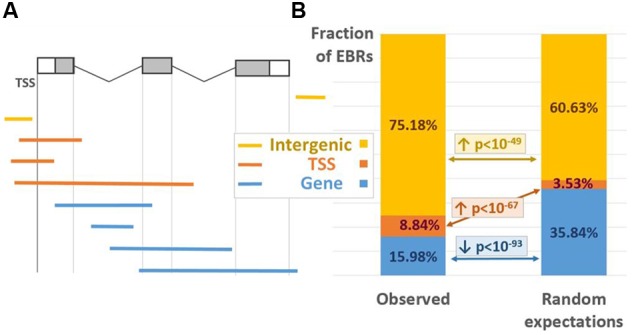
**EBRs distribution between various location classes. (A)** The positioning of EBRs corresponding to three location classes relative to the gene structure. White/gray boxes denote UTRs/exons; black lines denote introns. **(B)** Observed (left) and expected (right) fractions of the EBRs referred to three location classes. The *p*-values were derived from Monte-Carlo permutation test (Boldyreva et al., 2016; see Analysis of the Distribution of EBRs). The arrows up/down denote significant enrichment/depletion. Yellow/red/blue colors denote INTERGENIC/TSS/GENE location classes.

To evaluate the distribution of ChIP-Seq derived EBRs relative to the gene structure we distinguished three EBR location classes, modified from (Boldyreva et al., 2016). The “TSS” class contained EBRs overlapping with at least one of the gene TSSs; all other EBRs overlapping the gene bodies were attributed to the “GENE” class; EBRs falling outside of any genes were classified as “INTERGENIC” (**Figure 1A**). The major fraction of EBRs (75.18%) was classified as “INTERGENIC,” 15.98% were assigned to “GENE” class, 8.84% overlapped TSSs (**Figure 1B**, left). The enrichment of EBRs in three location classes relative to random expectations was statistically estimated implying permutation Monte-Carlo test (see Analysis of the Distribution of EBRs). The EBRs were distributed non-randomly relative to the gene structure (**Figure 1B**). The “TSS” and “INTERGENIC” classes were significantly enriched in EBRs (*p* < 1E-67 and *p* < 1E-49, correspondingly), whereas gene bodies showed notable underrepresentation of EBRs (*p* < 1E-93). In general, such a binding profile is quite common for TFs and consistent with their functions (Heyndrickx et al., 2014).

**FIGURE 2:**
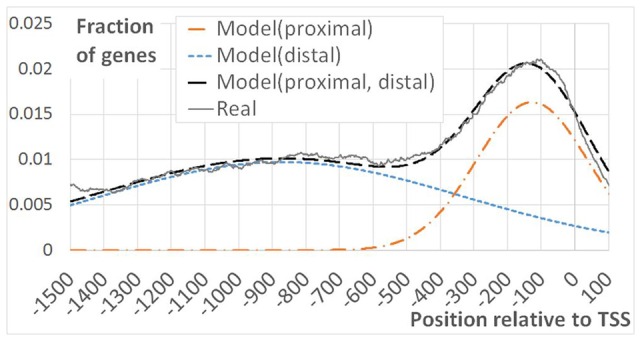
**Bimodal EBRs distribution in the upstream regions of *A. thaliana* genes.** The figure shows the fraction of genes mapped to EBRs at a certain distance from TSS as a function of the distance from TSS. 19434 genes with annotated 5′UTR were considered in the analysis. Model(proximal), Model(distal) – Gaussian distribution functions. Model_i_ = *A*_i_^∗^Norm(x,m_i_,σ_i_), (i = distal/proximal), x – the distance from TSS, m, σ –mean and standard deviation of the normal distribution. Model(proximal, distal) – the joined model, defined as the linear function. Model(proximal, distal) = C + Model(proximal)+Model(distal). Here C respects to the impact of non-specific DNA-EIN3 interactions. Real – observed distribution of ChIP-Seq derived EBRs. The constants C, *A*_i_, m_i_, σ_i_ were chosen empirically to provide the best approximation of the real curve “Real” by the model “Model (proximal, distal).”

**FIGURE 3:**
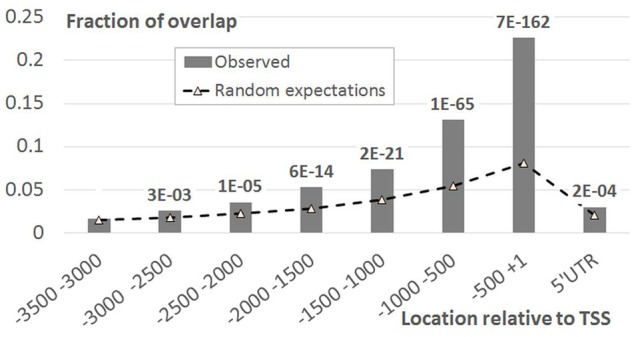
**EBRs distribution in the upstream regions of *A. thaliana* genes compared to random expectations.** *X*-axis denotes the genomic intervals relative to TSS, *Y*-axis – the ratio of total length of overlap between EBRs and a certain interval to the total length of EBRs. Data labels denote *p-*values derived from Monte-Carlo permutation test (Khoroshko et al., 2016, see Monte-Carlo Permutation Test for Genome Tracks).

As EBRs were overrepresented in genomic regions encompassing TSS and in intergenic spacers, we performed more detailed analysis of the [-1500; +100] upstream gene regions. Namely, for each position we calculated the fraction of genes overlapping EBRs (**Figure 2**). An evidently prevalent EBS location was found immediately upstream of the TSS in agreement with previously reported observations (Chang et al., 2013). Unexpectedly, we found that the distribution of EBRs was bimodal: the additional extended peak was observed in the distal upstream region (**Figure 2**). The enrichment of EBRs in the distal region was significant according to the permutation Monte-Carlo test up to -3000 relative to TSS (**Figure 3**). The bimodal distribution was not previously reported for EBRs. Thus, we questioned if the observed bimodality is essential for EIN3-mediated transcriptional regulation.

### Distribution of EBRs in Upstream Gene Regions in Different Chromatin States

The DNA binding by TFs is often guided by the chromatin state, which is supposed to have a conserved positional order (Filion et al., 2010; Sequeira-Mendes et al., 2014). To study the consistent patterns of epigenetic impact into the formation of two distinct EIN3 binding loci we appealed to previously published genome-wide map of nine chromatin states in *A. thaliana* (Sequeira-Mendes et al., 2014). The profile of EBRs distribution in the distal and proximal regions was in good accordance with the genome distribution of chromatin states 4 and 2, correspondingly (Supplementary Figure S4). This implied that the two modes of EBRs distribution could be associated with the distinct chromatin states. To clarify the epigenetic content of two peaks (distal and proximal) we characterized all EBRs with respect to the epigenetic status and analyzed their distribution in [-1500; +100] region separately. The EBRs were significantly overrepresented not only in the domains within mentioned above active chromatin states 2 and 4, but also within active chromatin state 1 (according to permutation Monte-Carlo test *p* < 1E-135, *p* < 1E-70, *p* < 1E-5, correspondingly). All the rest chromatin states were significantly depleted in EBRs.

**FIGURE 4:**
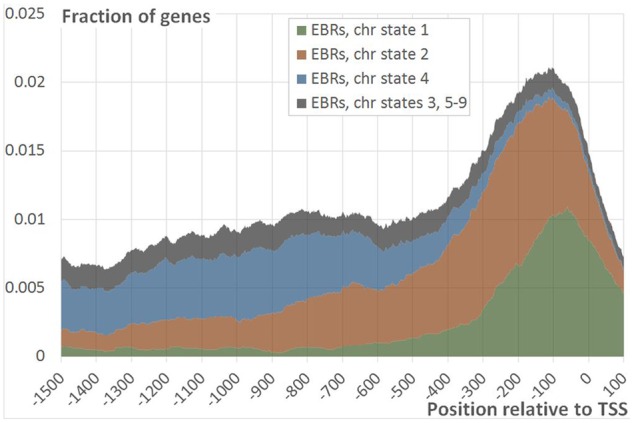
**Distribution of EBRs in different chromatin states.** The figure shows the fraction of the genes mapped to EBRs within the specific chromatin state at a certain distance from TSS as a function of the position relative to TSS. Summation of fractions of genes mapped to EBRs with chromatin states 1 (green), 2 (red), 4 (blue) and all the rest states (gray) gives the total distribution of EBRs relative to TSS (data row ‘Real’ on **Figure 2**).

**FIGURE 5:**
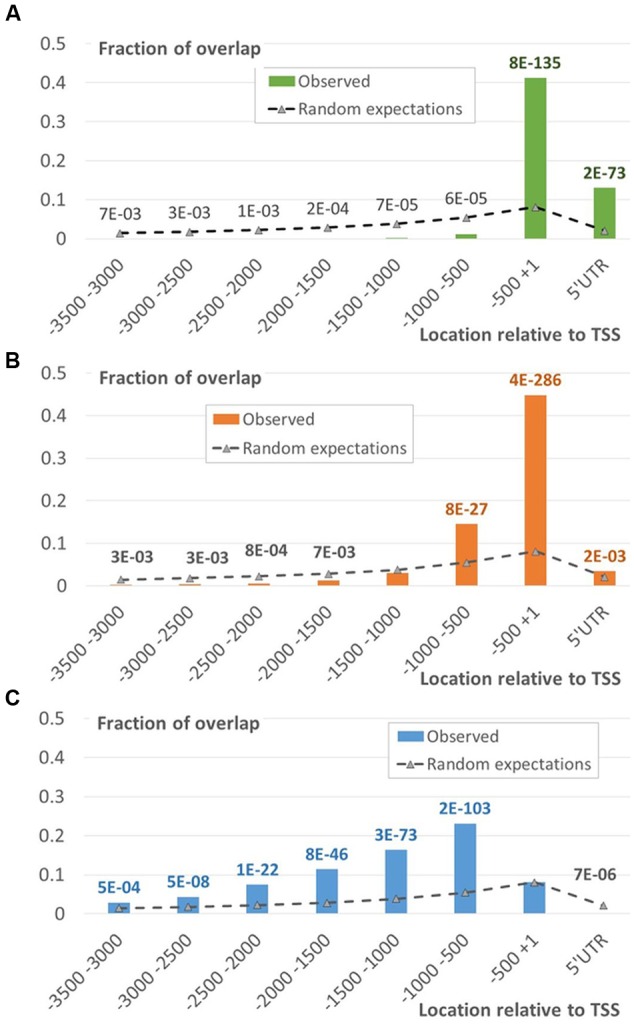
**Distribution of EBR fragments located within the chromatin states 1, 2 and 4.** The distributions of EBRs located in the domains of chromatin states 1 **(A)**, 2 **(B)**, or 4 **(C)** mapped to specific intervals of the upstream gene regions. *X*-axis denotes genomic intervals relative to TSS, *Y*-axis – the ratio of total length of overlap between EBR fragments and a certain interval to the total length of EBR fragments. Data labels denote *p*-values derived from the Monte-Carlo permutation test (Khoroshko et al., 2016; see Materials and Methods). Green/Blue/Red fonts of labels denote enrichment of the observed values respective to the expected ones, the gray font corresponds to depletion.

The EBRs related to two different chromatin states (states 1 and 2) formed the major fraction of the proximal peak in the EBRs distribution profile (**Figures 4** and **5A,B**; Supplementary Figure S5). The implicit distal peak predominantly consisted of EBRs mapped to the domains within chromatin state 4 (**Figure 4**), which was characterized by increased level of a repressive mark compared to the states 1 and 2 (Sequeira-Mendes et al., 2014). It is also noteworthy that non-random occurrence of the state 4 EBRs was statistically confirmed for the distal promoter region but not for the proximal one (permutation Monte-Carlo test, **Figure 5C**).

Thus, basically, the segregation of the distal peak in EBRs distribution could reflect the impact of the chromatin state 4 on EIN3 binding to DNA. In turn it might determine an alternative EIN3 regulation mode, which differs from the one implemented upon EIN3 binding to the proximal promoter region.

### Association of EBRs in Chromatin State 4 and Ethylene Response

To test if the EBRs located in different epigenetic context could be related to distinct EIN3 regulation modes, we performed GO enrichment analysis. We linked all EBRs to the neighboring genes if they overlapped with their [-3500; +1] upstream regions. For further functional analysis, we kept only genes univocally associated with either ‘state 1 or 2’ or ‘state 4’ EBRs [see Gene Ontology (GO) Enrichment Analysis for details] since the other states of chromatin were depleted in the whole EBRs dataset (see Distribution of EBRs in Upstream Gene Regions in Different Chromatin States). As a result, we obtained two list of 1117/601 genes harboring only ‘state 1 or 2’/‘state 4’ EBRs (Supplementary Tables S1 and S2 for respective data derived by the alternative procedure described in Supplementary Data 1). These lists of genes were analyzed separately with DAVID tool (Huang et al., 2009). Each GO term in output was estimated by a *p*-value and a fraction of involved genes. To filter the most robust GO terms, we required that the former is significant in terms of FDR < 0.05 according to Benjamini-Hochberg correction, and the latter was greater than 3%. We considered vocabularies of BP, MF, and CC and found 3/2/5 and 15/1/0 robust GO terms belonging to BP/MF/CC vocabularies enriched in the EBRs associated gene lists in ‘states 1 or 2’ and ‘state 4,’ correspondingly (Supplementary Tables S3 and S4 for respective data derived by the alternative procedure described in Supplementary Data 1).

**FIGURE 6:**
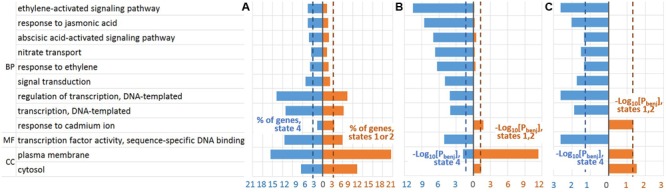
**Robust GO terms significantly enriched in the genes harboring EBRs within a specific chromatin state.** EBRs of chromatin state 4 were compared against those of ‘state 1 or 2’. **(A)** The fractions of involved genes. **(B)** FDR adjusted *p*-value that reflected enrichment of GO terms in the genes harboring EBRs in comparison with whole-genome expectation [see Gene Ontology (GO) Enrichment Analysis]. **(C)** FDR adjusted *p*-value that reflected the enrichment of GO terms in ‘state 4’ and ‘states 1 or 2’ gene lists [see Gene Ontology (GO) Enrichment Analysis]. BP, biological processes vocabulary; MF, molecular functions vocabulary; CC, cellular components vocabulary; *P*_benj_, FDR adjusted *p*-value (Benjamini-Hochberg procedure). Blue/red color indicates the corresponding values in ‘state 4’/‘state 1 or 2’ lists. The dotted lines denote the thresholds used to select the robust GO terms: 3% of involved genes; FDR adjusted *p-*value < 0.05.

To study if the robust GO terms specifically enriched for a certain gene list relative to the other one we performed additional Fisher’s exact test [see Gene Ontology (GO) Enrichment Analysis] (Supplementary Tables S3 and S4). Thus, we distinguished (1) nine GO terms significantly overrepresented in ‘state 4’ list in comparison to ‘states 1 or 2’ list and (2) three GO terms with the reverse enrichment (**Figure 6C**). It is noteworthy, that ethylene related GO terms were significantly enriched specifically in ‘state 4’ over ‘states 1 or 2’ list (**Figure 6**, Supplementary Tables S3 and S4). Wherein, among BP GO terms, *GO:0009873∼ethylene-activated signaling pathway* was most significantly enriched in ‘state 4’ list relative to both genome background and ‘state 1 or 2’ list (**Figures 6B,C**, Supplementary Tables S3 and S4). Besides, GO terms related to transcription activation were also found specifically enriched in ‘state 4’ over ‘states 1 or 2’ gene list.

Taken together, we conclude that EBRs in chromatin state 4 might play a role in general ethylene signaling. We find it noteworthy that chromatin state 4 is characterized by elevated amount of repressive mark H3K27me3 and a decreased number of active chromatin marks compared to states 1 and 2 (Sequeira-Mendes et al., 2014). Hence, an association of ethylene responsive elements with chromatin state 4 might indicate that EIN3 mediated transcription is epigenetically regulated by recruiting H3K27me3 demethylases (Gan et al., 2015). Previously, epigenetic regulation was reported for the ethylene-jasmonate crosstalk mediated by EIN3 (Zhu et al., 2011). Presumably, in other EBRs located in chromatin states 1 and 2, another mechanism of transcriptional regulation might be implemented. These findings extend our understanding of previously reported observations on EIN3 functioning. In (Chang et al., 2013) it was shown that only a minor fraction of ChIP-Seq derived EBRs (about 30%) is associated with altering gene expression upon ethylene treatment, whereas for majority of EBRs EIN3 binding is not sufficient for triggering gene expression implying that these loci function in integrating ethylene unrelated signals. Thus, we conclude that there is a spatial segregation of two mentioned types of EBRs caused (at least partially) by their epigenetic status.

### Enrichment of Putative EIN3 Binding Sites in Chromatin State 4

**FIGURE 7:**
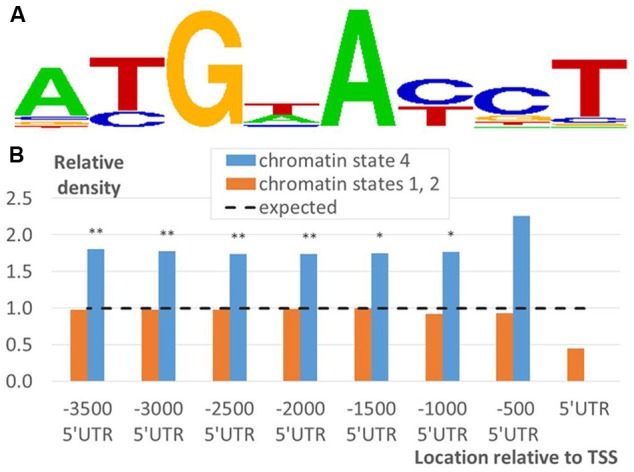
**Distribution of potential EBSs in EBRs mapped to the upstream regions of *A. thaliana* genes. (A)** Logo for TEIL motif, a binding site of EIN3 homolog in tobacco (Kosugi and Ohashi, 2000), computed by STAMP tool (Mahony and Benos, 2007). **(B)** Relative density of predicted EBSs in EBRs mapped in the domains of chromatin state 4 and 1/2 over a certain distance relative to TSS. *X*-axis denotes the location of the fragments relative to TSS. *Y*-axis denotes the densities of potential sites predicted by TEIL model **(A)**. The density was normalized to the respective average density computed for the whole genome. Labels ‘^∗^’ and ‘^∗∗^’ denote the Bonferroni’s corrected significance *p* < 0.05/8 and 0.01/8 estimated by Fisher’s exact test.

To investigate the influence of the genetic component on functional segregation of EBRs described in the previous section, we investigated distribution of potential EBSs in EBR fragments corresponding to the chromatin states 1, 2, or 4 (see Prediction of Putative EIN3 Binding Sites). To evaluate the occurrence of DNA sequences specifically recognized by EIN3 we modeled potential EBS using the PWM deduced from the position frequency matrix for TEIL motif (**Figure 7A**), the binding site of EIN3 homolog in tobacco (Kosugi and Ohashi, 2000).

We find that potential EBSs were significantly enriched in ‘state 4’ EBR fragments relative to ‘state 1 or 2’ ones according to Fisher’s exact test (**Figure 7B**; Supplementary Figure S6). Thus, taking into account EBRs distribution with respect to the chromatin states (**Figures 4** and **5**; Supplementary Figure S5) the implicit distal peak was significantly associated not only with ethylene response function, but also with the specific DNA context recognized by EIN3. Intriguingly, the pronounced proximal peak was not associated with neither of them. This result implies two important conclusions. First, it supports the idea of a role of epigenetic regulation in EIN3 mode of functioning, which mechanisms are to be elucidated in future studies. Second, the high density of ChIP-Seq derived binding regions does not univocally prescribe overrepresentation of the specific DNA context. Particularly, this highlights an alternative mode of functioning for the majority of proximal EBRs, which is also a subject for further investigations.

## Conclusion

ETHYLENE-INSENSITIVE3 is the master transcriptional regulator of ethylene signaling. It acts as a hub that integrates plant signals and redistributes them triggering distinct transcriptional responses to adapt to volatile environment. We found that EBRs in upstream gene regions have a bimodal distribution with a pronounced proximal peak and a broad implicit distal peak, both significant relative to random expectations. The EBRs were significantly overrepresented only in the domains within chromatin states 1, 2, and 4. We predicted an importance of chromatin state 4 both in formation of the distal peak and in EIN3 regulation of ethylene response. The latter statement is supported by GO enrichment analysis. Moreover, we showed that the potential EBSs are associated with the chromatin state 4, but not 1 or 2. Such a profile provides the idea of interplay between genetic and epigenetic factors, which may determine at least two distinct modes of EIN3 regulation mediated by spatially separated EBRs.

## Author Contributions

EZ initiated, designed, and coordinated the study, interpreted the results, and wrote the manuscript. VL and DO analyzed the data. VL, DO, IG, and VM participated in the design of the study, the interpretation of the results, and the revision of the manuscript. All authors read and approved the final manuscript.

## Conflict of Interest Statement

The authors declare that the research was conducted in the absence of any commercial or financial relationships that could be construed as a potential conflict of interest.

## Footnotes

1 https://www.ncbi.nlm.nih.gov/sra/

2 ftp://ftp.arabidopsis.org/home/tair/Sequences/whole_chromosomes/

3 https://www.encodeproject.org/data-standards/chip-seq/

4 ftp://ftp.arabidopsis.org/Maps/gbrowse_data/TAIR10/TAIR10_GFF3_genes.gff

5 https://david.abcc.ncifcrf.gov/

## Abbreviations

EBR: EIN3 binding region
EBS: EIN3 binding site
EIL: ETHYLENE-INSENSITIVE3-LIKE
EIN3: ETHYLENE-INSENSITIVE3
GO: Gene Ontology
TEIL: TOBACCO EIN3-LIKE
TF: transcription factor
TSS: transcription start site

## References

[B1] Alonso J. M. Stepanova A. N. Solano R. Wisman E. Ferrari S. Ausubel F. M. (2003). Five components of the ethylene-response pathway identified in a screen for weak ethylene-insensitive mutants in *Arabidopsis*. *Proc. Natl. Acad. Sci. U.S.A.* 100 2992–2997. 10.1073/pnas.0438070100 12606727 PMC151454

[B2] An F. Zhang X. Zhu Z. Ji Y. He W. Jiang Z. (2012). Coordinated regulation of apical hook development by gibberellins and ethylene in etiolated *Arabidopsis* seedlings. *Cell Res.* 22 915–927. 10.1038/cr.2012.29 22349459 PMC3343656

[B3] An F. Zhao Q. Ji Y. Li W. Jiang Z. Yu X. (2010). Ethylene-induced stabilization of ETHYLENE INSENSITIVE3 and EIN3-LIKE1 is mediated by proteasomal degradation of EIN3 binding F-Box 1 and 2 that requires EIN2 in *Arabidopsis*. *Plant Cell* 22 2384–2401. 10.1105/tpc.110.076588 20647342 PMC2929093

[B4] Benjamini Y. Hochberg Y. (1995). Controlling the false discovery rate: a practical and powerful approach to multiple testing. *J. R. Stat. Soc. B. Methodol.* 57 289–300.

[B5] Boldyreva L. Goncharov F. Demakova O. Zykova T. Levitsky V. Kolesnikov N. (2016). Protein and genetic composition of four basic chromatin types in *Drosophila melanogaster* cell lines. *Curr. Genomics* 10.2174/1389202917666160512164913 [Epub ahead of print]. PMC5345337 28367077

[B6] Chang K. N. Zhong S. Weirauch M. T. Hon G. Pelizzola M. Li H. (2013). Temporal transcriptional response to ethylene gas drives growth hormone cross-regulation in *Arabidopsis*. *Elife* 2:e00675 10.7554/eLife.00675 PMC3679525 23795294

[B7] Chao Q. Rothenberg M. Solano R. Roman G. Terzaghi W. Ecker J. R. (1997). Activation of the ethylene gas response pathway in *Arabidopsis* by the nuclear protein ETHYLENE-INSENSITIVE3 and related proteins. *Cell* 89 1133–1144. 10.1016/S0092-8674(00)80300-1 9215635

[B8] Cho Y. H. Yoo S. D. (2015). Novel connections and gaps in ethylene signaling from the ER membrane to the nucleus. *Front. Plant Sci.* 5:733 10.3389/fpls.2014.00733 PMC4283510 25601870

[B9] Filion G. J. van Bemmel J. G. Braunschweig U. Talhout W. Kind J. Ward L. D. (2010). Systematic protein location mapping reveals five principal chromatin types in *Drosophila* cells. *Cell* 143 212–224. 10.1016/j.cell.2010.09.009 20888037 PMC3119929

[B10] Gan E. S. Xu Y. Ito T. (2015). Dynamics of H3K27me3 methylation and demethylation in plant development. *Plant Signal. Behav.* 10:1027851 10.1080/15592324.2015.1027851 PMC4883920 26313233

[B11] Guo H. Ecker J. R. (2004). The ethylene signaling pathway: new insights. *Curr. Opin. Plant. Biol.* 7 40–49. 10.1016/j.pbi.2003.11.011 14732440

[B12] Heyndrickx K. S. Van de Velde J. Wang C. Weigel D. Vandepoele K. (2014). A functional and evolutionary perspective on transcription factor binding in *Arabidopsis thaliana*. *Plant Cell* 26 3894–3910. 10.1105/tpc.114.130591 25361952 PMC4247581

[B13] Huang D. W. Sherman B. T. Lempicki R. A. (2009). Systematic and integrative analysis of large gene lists using DAVID bioinformatics resources. *Nat. Protoc.* 4 44–57. 10.1038/nprot.2008.211 19131956

[B14] Khoroshko V. A. Levitsky V. G. Zykova T. Y. Antonenko O. V. Belyaeva E. S. Zhimulev I. F. (2016). Chromatin heterogeneity and distribution of regulatory elements in the late-replicating intercalary heterochromatin domains of *Drosophila melanogaster* chromosomes. *PLoS ONE* 11:e0157147 10.1371/journal.pone.0157147 PMC4907538 27300486

[B15] Kosugi S. Ohashi Y. (2000). Cloning and DNA-binding properties of a tobacco Ethylene-Insensitive3 (EIN3) homolog. *Nucleic Acids Res.* 28 960–967. 10.1093/nar/28.4.960 10648789 PMC102569

[B16] Langmead B. Trapnell C. Pop M. Salzberg S. L. (2009). Ultrafast and memory-efficient alignment of short DNA sequences to the human genome. *Genome Biol.* 10:R25 10.1186/gb-2009-10-3-r25 PMC2690996 19261174

[B17] Levitsky V. G. Ignatieva E. V. Ananko E. A. Turnaev I. I. Merkulova T. I. Kolchanov N. A. (2007). Effective transcription factor binding site prediction using a combination of optimization, a genetic algorithm and discriminant analysis to capture distant interactions. *BMC Bioinformatics* 8:481 10.1186/1471-2105-8-481 PMC2265442 18093302

[B18] Mahony S. Benos P. V. (2007). STAMP: a web tool for exploring DNA-binding motif similarities. *Nucleic Acids Res.* 35 W253–W258. 10.1093/nar/gkm272 17478497 PMC1933206

[B19] McManus M. T. (2012). *The Plant Hormone Ethylene. Annual Plant Reviews.* Oxford: Wiley-Blackwell 10.1002/9781118223086

[B20] Merchante C. Alonso J. M. Stepanova A. N. (2013). Ethylene signaling: simple ligand, complex regulation. *Curr. Opin. Plant Biol.* 16 554–560. 10.1016/j.pbi.2013.08.001 24012247

[B21] Sequeira-Mendes J. Aragüez I. Peiró R. Mendez-Giraldez R. Zhang X. Jacobsen S. E. (2014). The functional topography of the *Arabidopsis* genome is organized in a reduced number of linear motifs of chromatin states. *Plant Cell* 26 2351–2366. 10.1105/tpc.114.124578 24934173 PMC4114938

[B22] Solano R. Stepanova A. Chao Q. Ecker J. R. (1998). Nuclear events in ethylene signaling: a transcriptional cascade mediated by ETHYLENE-INSENSITIVE3 and ETHYLENE-RESPONSE-FACTOR1. *Genes Dev.* 12 3703–3714. 10.1101/gad.12.23.3703 9851977 PMC317251

[B23] Song S. Huang H. Gao H. Wang J. Wu D. Liu X. (2014). Interaction between MYC2 and ETHYLENE INSENSITIVE3 modulates antagonism between jasmonate and ethylene signaling in *Arabidopsis*. *Plant Cell* 26 263–279. 10.1105/tpc.113.120394 24399301 PMC3963574

[B24] Zhang Y. Meyer C. A. Eeckhoute J. Johnson D. S. Bernstein B. E. Nusbaum C. (2008). Model-based analysis of ChIP-Seq (MACS). *Genome Biol.* 9:R137 10.1186/gb-2008-9-9-r137 PMC2592715 18798982

[B25] Zhong S. Shi H. Xue C. Wang L. Xi Y. Li J. (2012). A molecular framework of light-controlled phytohormone action in *Arabidopsis*. *Curr. Biol.* 22 1530–1535. 10.1016/j.cub.2012.06.039 22818915 PMC4437768

[B26] Zhu Z. An F. Feng Y. Li P. Xue L. Mu A. (2011). Derepression of ethylene-stabilized transcription factors (EIN3/EIL1) mediates jasmonate and ethylene signaling synergy in *Arabidopsis*. *Proc. Natl. Acad. Sci. U.S.A.* 108 12539–12544. 10.1073/pnas.110395910 21737749 PMC3145709

