# Supplementary material for: The Interplay of Chromatin Landscape and DNA-Binding Context Suggests Distinct Modes of EIN3 Regulation in Arabidopsis thaliana
Source: Front Plant Sci. 2017 Jan 9;7:2044. doi: 10.3389/fpls.2016.02044 (PMC5220190; doi:10.3389/fpls.2016.02044)
Supplement: Supplementary file 2 [file Data_Sheet_1.pdf]

Supplementary Material

The Interplay of Chromatin Landscape and DNA-Binding Context Suggests Distinct Modes of EIN3 Regulation in *Arabidopsis thaliana*

Elena V. Zemlyanskaya*, Victor G. Levitsky, Dmitry Y. Oshchepkov, Ivo Grosse, Victoria V. Mironova

*** Correspondence:** Elena V. Zemlyanskaya: ezemlyanskaya@bionet.nsc.ru

1 Supplementary Data 1

ChIP-Seq data analysis

Two publicly available raw ChIP-Seq datasets on EIN3 binding in three-days-old *A. thaliana* etiolated seedlings (Chang *et al.*, 2013) were retrieved from the NCBI Sequence Read Archive (SRA, http://www.ncbi.nlm.nih.gov/sra/). The dataset SRX216234 represents data on plants treated by ethylene for four hours at an ethylene gas concentration of 10 µl/l, and the dataset SRX215430 represents data on control plants without ethylene treatment. In addition, the publicly available dataset SRX398442 was retrieved from NCBI SRA as a negative control because the four ChIP-seq datasets on EIN3 binding lacked input DNA or mock-treated ChIP-seq control samples. The raw data of these three datasets were trimmed and quality-filtered with Trimmomatic v.0.36 (Bolger et al., 2014) to remove adapters, low quality bases at read ends, and low quality reads. Individual ChIP-Seq runs were pooled for each dataset.

The genome sequence of *A. thaliana* was retrieved from TAIR 10 (ftp://ftp.arabidopsis.org/home/tair/Sequences/whole_chromosomes/). The trimmed and quality-filtered ChIP-Seq reads were mapped to the genome sequence with Bowtie v. 1.1.1 (Langmead et al., 2009) using only unique alignments with no mismatches (-n 0 -m 1 --best). Peaks were called separately for two datasets on EIN3 binding with the callpeak function of MACS v. 2.1.1.20160309 (https://github.com/taoliu/MACS; Zhang et al., 2008) using the external dataset SRX398442 as a negative control. The peaks called in datasets for treated and control plants were compared with the bdgdiff function of MACS.

References

Bolger, A.M., Lohse, M., Usadel B. (2014). Trimmomatic: a flexible trimmer for Illumina sequence data. *Bioinformatics*. 30, 2114-2120. doi: 10.1093/bioinformatics/btu170 PMCID: PMC4103590

2 Supplementary Figures

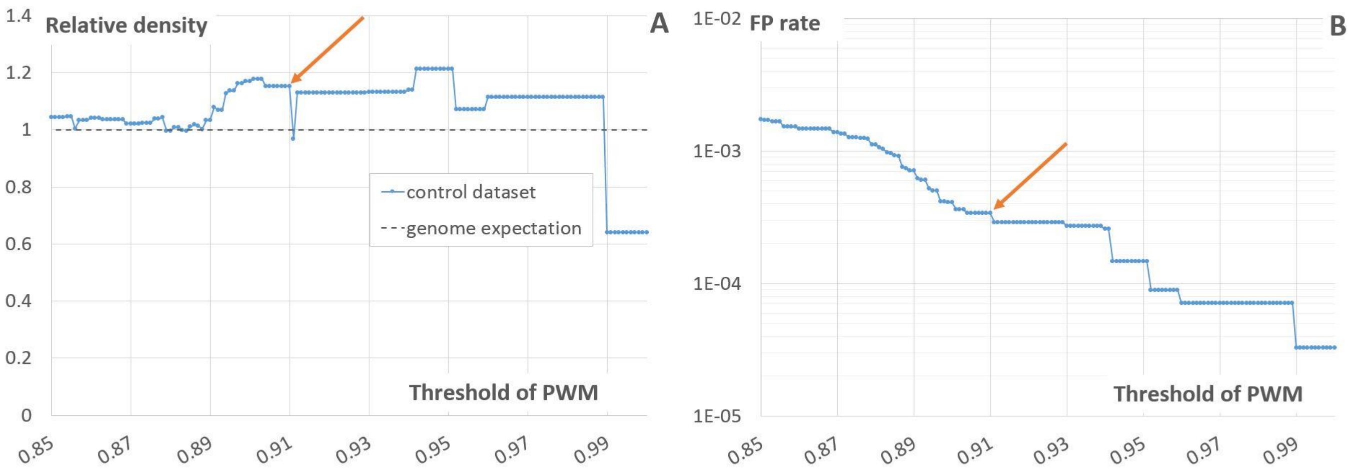

Figure S1. Threshold selection for the TEIL PWM model. Panel **(A)** shows the relative density of EBS predicted in the control dataset as a function of the threshold of the TEIL PWM model. Panel **(B)** shows the False Positive (FP) rate as a function of the threshold of the TEIL PWM model. FP rate is computed as the recognition rate for the whole genome. The threshold of 0.91 (denoted by arrows) was selected for further analysis as it is located in the beginning of the plateau of relative density (0.9-1) with the recognition rate for the control dataset 15% more than the whole genome expectation and possesses the permissive FP rate of 3.4E-4. Increasing the threshold will leads to the increase of False Negative rate as EIN3 binding site is poorly conserved (Kosugi and Ohashi, 2000).

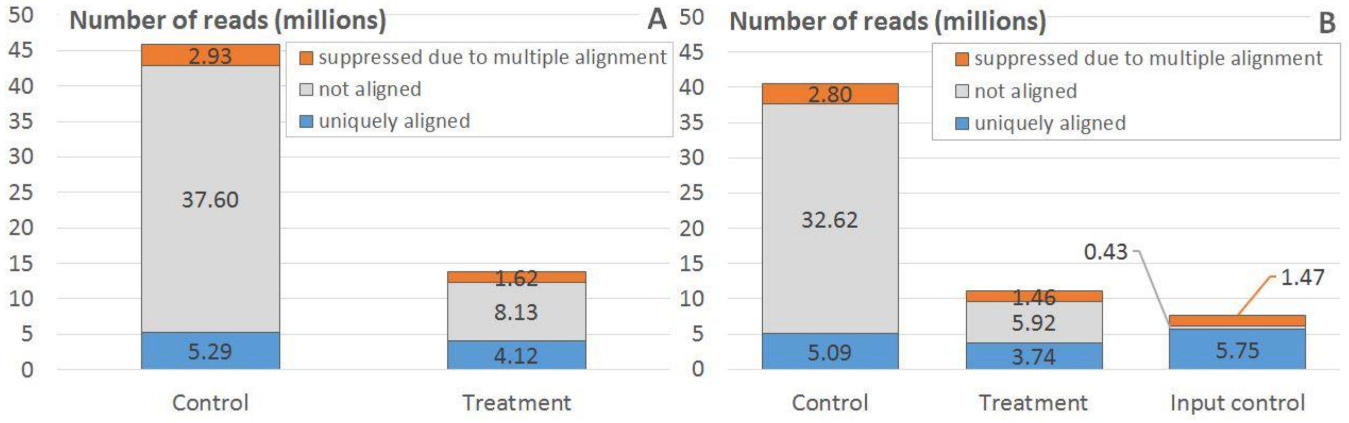

Figure S2. Mapping quality of ChIP-seq samples. (A) Number of reads in the alignments of control/treated datasets SRX215430/SRX216234, which were processed by the procedure described in Materials and Methods. **(B)** Number of reads in the alignment of the same datasets and input control dataset SRX398442, which were processed by the procedure described in Supplementary Data 1.

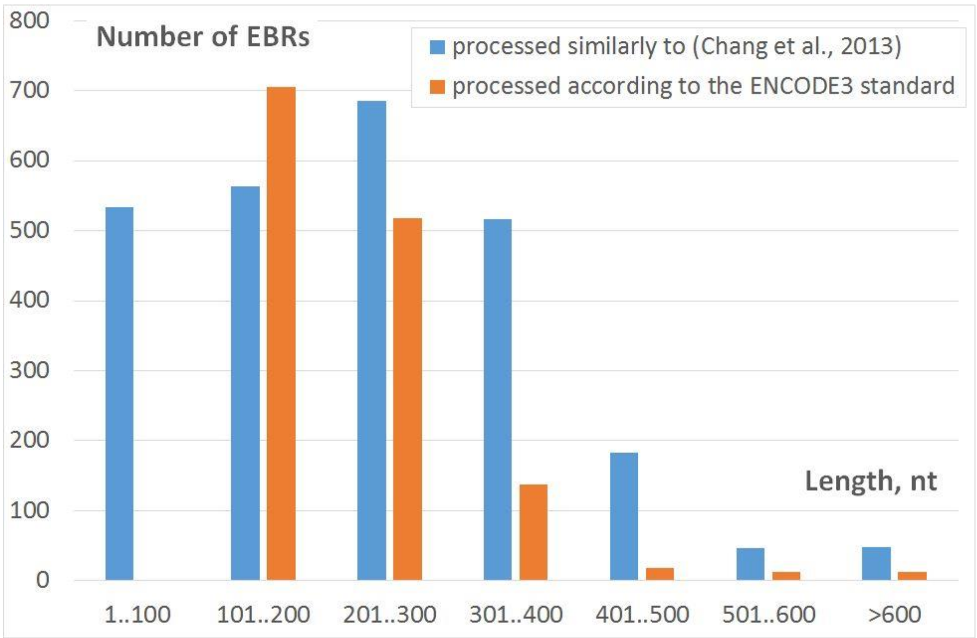

Figure S3. Length distribution of ChIP-Seq derived EBRs. The figure shows the number of EBRs with the certain length as a function of EBR length intervals. The distributions for ChIP-Seq data processed similarly to (Chang et al., 2013) (see Materials and Methods) and according to the ENCODE3 standard (see Supplementary Data 1) are compared. Corresponding genome profiles in WIG/BED formats are represented in Supplementary data 2. In both cases, the majority of peaks (> 95%) had the length below 500 nucleotides.

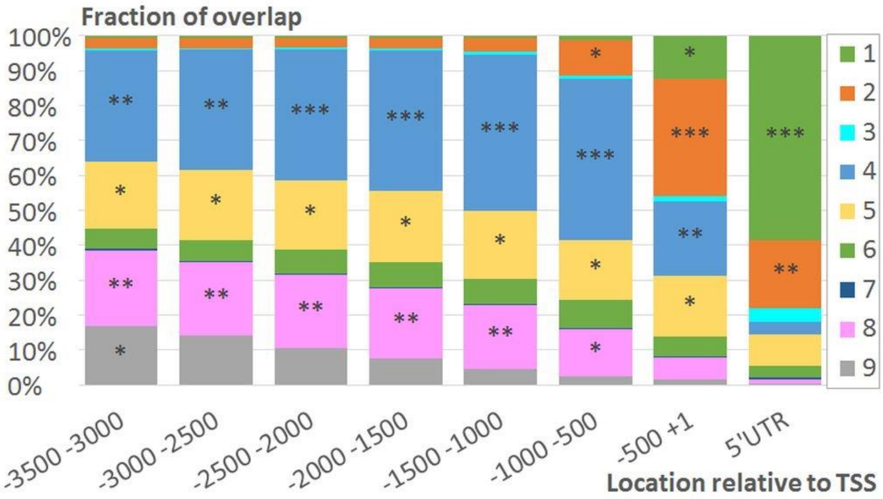

Figure S4. Distribution of chromatin states. The figure shows the ratio of total length of overlap between chromatin domains and a certain interval to the total length of chromatin domains as a function of the genomic intervals relative to TSS. The labels '*', '**' and '***' denote significant differences from random expectations according to the Monte-Carlo permutation test (Khoroshko et al., 2016) with *p*-values in the ranges $p < 0.05/72$, $p <$ 1E-100 and $p <$ 1E-500, correspondingly. States 2 and 4 possess their maximum in upstream gene regions.

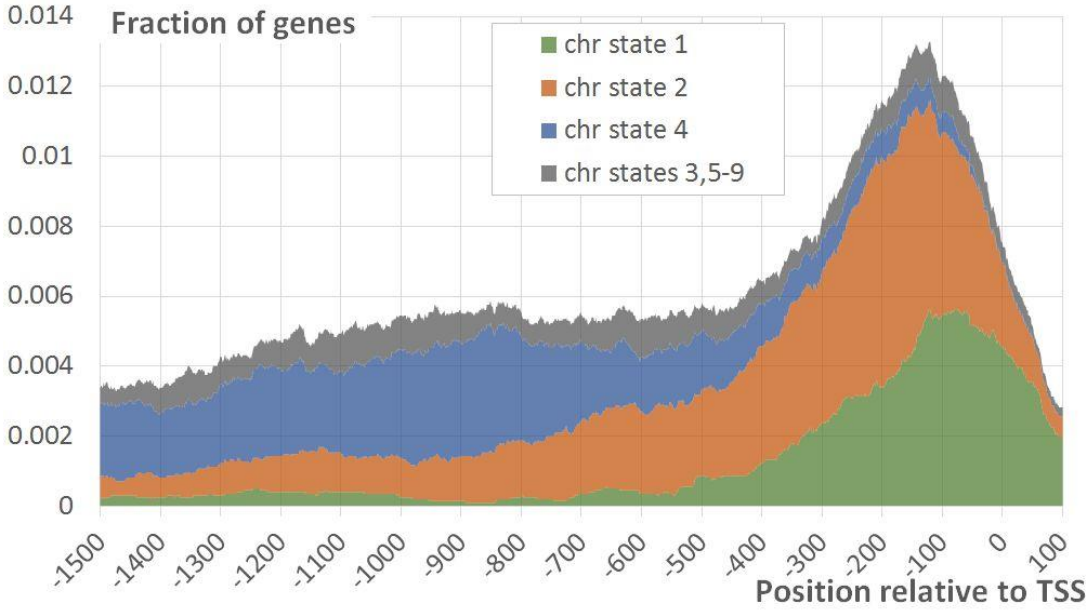

Figure S5. Distribution of EBRs in different chromatin states. The figure shows the fraction of the genes mapped to EBRs within the specific chromatin state at a certain distance from TSS as a function of the position relative to TSS. Raw ChIP-Seq data were processed by the procedure described in Supplementary Data 1.

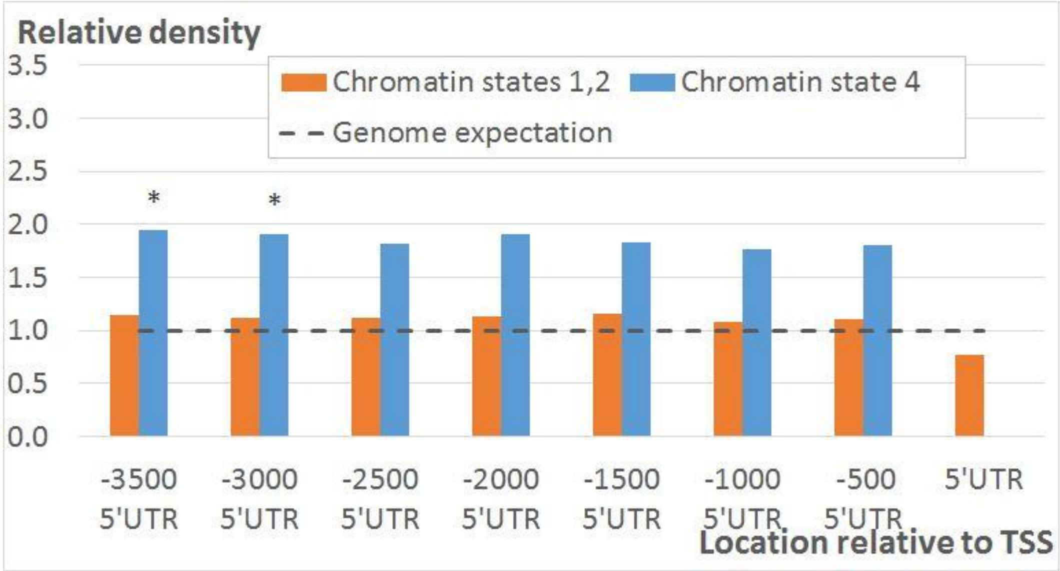

Figure S6. Relative density of predicted EBSs in EBRs mapped in the domains of chromatin states 4 and 1/2. The figure shows the densities of potential sites predicted in EBRs by TEIL model as a function of the location of the fragments relative to TSS. Raw ChIP-Seq data were processed by the procedure described in Supplementary Data 1. The density was normalized to the respective average density computed for the whole genome. Labels '*' and '**' denote the Bonferroni's corrected significance $p < 0.05/8$ and 0.01/8 estimated by Fisher's exact test.
